# Recent trend and correlates of induced abortion in China: evidence from the 2017 China Fertility Survey

**DOI:** 10.1186/s12905-022-02074-5

**Published:** 2022-11-24

**Authors:** Tian Wang, Quanbao Jiang

**Affiliations:** School of Public Policy and Administration, Institute for Population and Development Studies, Xi’an Jiaotong University, Xi’an, China

**Keywords:** Induced abortion, Repeat induced abortion, Sex-selective abortion, China

## Abstract

**Background:**

Although there are more than 10 million induced abortions per year in China, there are few comprehensive, systematic, and characteristic-based data on induced abortions among Chinese women. This study aims to examine the overall trend in induced abortions in China and to analyze the correlation between induced abortions and some socio-economic factors.

**Methods:**

Drawing from the 2017 China Fertility Survey, this study analyzed induced abortions using multiple indicators from period and cohort perspectives on a sample of 240,957 women. The indicators include the abortion rate and proportion, average age at the time of induced abortion, age-specific cumulative proportions, and the number of induced abortions by cohort. The analysis also differentiated based on residency, ethnicity, education level, and marital status. A binomial logistic regression model was used to examine the association between induced abortions and socio-economic factors.

**Results:**

Between 2006 and 2016, among women aged 15–49, there was an increase in the induced abortion rate and the average age of women who had induced abortions, but a decline in the proportion of abortions. The proportion of induced abortion was higher among premarital than post-marital pregnancies, among unintended than planned pregnancies. Women with induced abortion experiences accounted for less than 30% of all cohorts, and the cumulative number of induced abortions per woman in each cohort was less than 0.45. These indicators varied with birth cohort, residence, ethnicity, education level, and marital status. The results of binomial logistic regression confirmed the association between induced abortion and these socio-economic variables. Sex-selective abortions of female fetuses still exist, despite the government’s considerable efforts to eliminate them.

**Conclusion:**

The practice of induced abortions differs by cohort and socio-economic characteristics. The profile of women who resort to abortions in China has shifted from well-educated urban women to rural, less-educated women. More effective measures should be taken by the government to reduce the number of induced abortions among women with higher abortion risks.

## Introduction

A large number of induced abortions occur worldwide. In 2003, the total number of induced abortions was 42 million globally, and the induced abortion rate was 29 per 1000 women aged 15–44 years [1]. Between 2015 and 2019, the rate was 39 per 1000 women aged 15–49 years, with an average of 73 million induced abortions per year [2]. A large proportion of induced abortions occur in China [3]. In 2019, the number of induced abortions documented in China was 9.76 million [4]. However, given the underreporting and concealment of induced abortions that occur in private hospitals and clinics, the actual number is likely to be higher. The actual number of induced abortions per year was estimated at approximately 13 million [5]. According to a recent report by the United Nations Population Fund, almost half of all pregnancies are unintended, and many end in induced abortions [6]. Reducing unintended pregnancies and, by extension, induced abortions would entail improving women’s sexual and reproductive health through contraceptive services and access to sex education [7, 8]. There has been a concerted effort in this regard from the government, academia, and the public to reduce induced abortion in China.

Previous research has shown that induced abortions are associated with individual characteristics and socio-economic factors [9–11]. First, age is an important factor: the average number of induced abortions increases with age [12], and high rates of repeat induced abortions are also associated with age [13, 14]. Second, residence status is another factor associated with induced abortion. China is characterized by clear urban-rural differences in many areas, including access to healthcare and birth control policies. While the One-child Policy was strictly enforced among urban residents, it was adjusted to a “1.5-child policy” for rural residents (rural couples whose first child was a girl were permitted to have another child) [15, 16]. Such policy variations have affected behaviors on fertility and induced abortion among women in urban and rural areas. Third, ethnicity is associated with women’s induced abortion in China [9, 10, 16]. A more relaxed birth control policy was implemented for ethnic-minority groups than for Han Chinese, causing more Han women to limit childbearing through induced abortions to conform to the implemented policy [11, 17].

Education level also affects women’s induced abortion behaviors in China [9, 11, 17], although research conclusions diverge in this area. Some studies have shown that less-educated women have less knowledge of reproductive health and contraception and are more likely to undergo induced abortion and repeat induced abortions [12, 18, 19]. However, other studies have shown that women with higher levels of education have higher abortion rates [9]. Women with more education are more likely to postpone childbearing, use short-term contraceptive methods, and resort to induced abortion if that fails.

Women’s marital status and sex of existing children are also associated with induced abortion [14, 15]. Married women and women with children are more likely to undergo repeat induced abortions in China [12]. In the past, induced abortions have generally occurred among married women constrained by the birth control policy. However, in recent times, abortions of premarital pregnancies have become widespread [20, 21]. Given the deeply-entrenched son preference and constraints on the number of births, many couples have turned to sex-selective abortions. If the first child is a girl, parents who prefer sons are likely to abort a second female fetus in pursuit of a son, resulting in a high rate of induced abortions after the first birth [16].

The large-scale induced abortions in China have attracted the attention of all sectors of society. However, most recent studies have used data drawn from small-scale surveys of women who had induced abortions in hospitals. This study analyzed the overall trend in induced abortions using a nationally representative sample. The analyses explore differences based on the birth cohort and individual characteristics. In addition, we examine the association between induced abortions and some correlates using a binomial logistic regression model. We aimed to provide a comprehensive period and cohort dataset for induced abortions among Chinese women and re-examine the factors that correlate with induced abortion.

## The Chinese context

### The birth control policy

Induced abortions are associated with China’s birth control policy. A stringent birth control policy was introduced in 1980, although enforcement was sometimes relaxed [15]. Many provincial family-planning regulations resort to induced abortion as a remedial measure for pregnancies out of the birth limit [22].

There were urban-rural differences in the implementation of the birth control policy. While couples with the urban Hukou-type (household registration) were permitted to have one child, rural Hukou-type couples in 19 provinces were allowed to have a second birth if the first child was a girl [15, 16]. Furthermore, there were differences in how the birth policy applied to Han citizens, as opposed to ethnic-minority groups. China is a multi-ethnic country with 56 ethnic groups. In the 2020 census, the Han people accounted for 91.11% of the population, while the other 55 ethnic groups accounted for approximately 9%. For many years, the Chinese government’s birth control policy was more relaxed for ethnic-minority groups than for the Han people. Consequently, the prevalence of induced abortion was higher among Han women [11, 17].

The stringent birth control policy has been gradually relaxed. In 2013, a selective two-child policy was adopted in which couples were permitted a second birth if either spouse was an only child. However, only 13.2% (1.45 million/11 million) of eligible couples had applied for permission to have a second child by May 2015 [23]. In 2016, a universal two-child policy was implemented for all citizens, regardless of Hukou-type or ethnicity. In 2021, China adopted a universal three-child policy. It remains to be seen how this policy relaxation will affect the trend of induced abortion [24].

### Premarital pregnancy

Premarital pregnancy is widely frowned upon in China. Influenced by traditional Confucianism, Chinese society values female virginity and denounces premarital sex [20, 25]. Owing to guilt and anxiety, some never-married young women who became pregnant were more likely to have induced abortions in private clinics or nonprofessional facilities to keep their families from knowing [26–28].

During the one-child policy era, out-of-wedlock births were labeled as “unauthorized” births and were ineligible for Hukou (household) registration. In China, many benefits and social welfare provisions are closely linked to the Hukou registration system [29, 30]. People without Hukou registration cannot access education, the medical system, or other state welfare provisions. Therefore, premarital pregnancies are usually terminated.

Recently, premarital cohabitation and sex have become widespread in China, but premarital childbearing is still considered unacceptable and it is uncommon. In 2018, the overall percentage of births registered with unmarried mothers was 41% in Organization for Economic Co-operation and Development (OECD) countries [31], but remarkably low in China [32]. The proportion of women who gave birth before marriage in different birth cohorts in China slightly increased from 0.2% among women born before 1974 to 1.2% in recent birth cohorts born between 1980 and 1989. Almost all children are still born and raised within marriage in contemporary China [32, 33]. Premarital pregnancies generally end in abortions, and unmarried women account for more than one-third of women undergoing induced abortions. Both the rate of induced abortion and the proportion of repeat induced abortions among young unmarried women continue to increase annually [34, 35].

### Sex-selective abortion

Sex-selective abortion also affects induced abortion in China. Given the strong preference for sons, some couples, especially in rural areas, turn to sex identification and selectively abort female fetuses to ensure a son within the birth control constraints [11, 16, 17, 36]. The likelihood of induced abortion is significantly higher for women with only daughters [16]. One data analysis showed that more than 25% of female fetuses ended in induced abortion, compared to 1.6% of male fetuses [37].

As gender equality has become mainstream, with education for women leading to increasing social status, the preference for sons is markedly waning [16]. When the universal two-child policy was implemented in 2016, sex-selective abortion decreased significantly, as manifested in the decline of China’s sex ratio at birth (expressed as the number of live male births for 100 live female births) [23, 38].

## Materials and methods

### Data

The data used in this study were drawn from two sources. Data on annual induced abortions between 1980 and 2020 were obtained from the *China Health Statistical Yearbook*, an annual statistical publication that documents developments in China’s public health and the health status of residents. Data on the annual number of births, drawn from the *China Statistical Yearbook*, were calculated based on the total population and birth rate. The *China Statistical Yearbook* is an annual statistical publication that details China’s economic and social situation, covering population, employment, government finances, prices, and agriculture, among other topics.

Other data (including the main data), were collected from the 2017 China Fertility Survey, conducted by the former National Health and Family Planning Commission. This survey was conducted with a female population aged 15–60. Through stratified three-stage probability proportional to size (PPS) sampling, the final valid sample size was 243,951. The survey was conducted in two ways: through face-to-face interviews and network surveys. A computer-assisted personal interviewing system was used in the questionnaire design, personnel training, sampling frame preparation, sampling, household survey, and questionnaire review. The post-enumeration quality check, comparison, and verification of the case data showed that the survey was highly accurate [39]. From these data, we excluded people over 60 years of age (n = 2380, 0.98%), those whose marriage/cohabitation information was missing (n = 23, 0.01%), and those whose age at first marriage or first pregnancy was under 15 (n = 591, 0.24%). This produced a final sample of 240,957 women. Unless otherwise specified, the data were sourced from the 2017 China Fertility Survey.

The survey includes the women’s pregnancy history, such as the end date of each pregnancy and result, categorized as live male birth, live female birth, stillbirth, spontaneous abortion, or induced abortion (including medical abortion and induced labor). Regardless of marital status, the interviewees completed a “pregnancy history,” specifying whether each pregnancy was planned.

### Methods

Various indicators were calculated to illustrate the overall trend and sample characteristics from a period and cohort perspective. Next, we conducted binomial logistic regression analysis to examine the association between induced abortions and correlates. All analyses were performed using the Stata version 15.

#### Period and cohort analysis

The indicators used for the induced abortion analysis included the number, rate, and proportion of induced abortions, as well as the ratio of births to abortions. Indicators, including the age-specific abortion rate and total abortion rate, were also applied [1, 9, 10]. In addition to period indicators, cohort indicators were also calculated, covering the rate and proportion of induced abortions. The main indicators of this study are as follows:

The *induced abortion rate* is defined as the number of induced abortions per 1000 women of childbearing age during a specified period (usually 1 year). Many studies in Western countries define women of childbearing age as 15–44 years old. [1, 3] Some studies in China use a range of 15–49 years old [9, 36]. In this study, we define childbearing age as 15–49 years old.

The *proportion of induced abortions* was defined as the proportion of pregnant women who experienced induced abortions during a specified period. Pregnant women included all women currently pregnant, as well as those who had experienced live male or female births, stillbirths, or induced abortions.

The *age-specific cumulative proportion of induced abortion for a cohort* refers to the proportion of all women in a cohort who have experienced at least “n” abortions by the corresponding age. For example, the “age-specific cumulative proportion of first abortion in a cohort” refers to the cumulative proportion of women in a cohort who have experienced at least one abortion by the corresponding age. This indicator can be calculated by residence, ethnicity, and education level.

The *age-specific cumulative number of induced abortions per woman* refers to the average number of abortions experienced by women in a cohort by the corresponding age. The numerator is the number of abortions experienced by women in a cohort by the corresponding age, and the denominator is the total number of women in the cohort. This also can be calculated by different factors.

Respondents were grouped into nine cohorts according to their age at the time of the survey. The cohorts were as follows: 15–19, 20–24, 25–29, 30–34, 35–39, 40–44, 45–49, 50–54, and 55–60. Due to the limited space, we used 25–29 or 30–34 cohorts, (women aged 25–29 or 30–34 at survey time) to represent the later birth cohort, and the 45–49 cohort (women aged 45–49 at survey time) to represent women in the earlier birth cohort.

We divided pregnancies into premarital and post-marital pregnancies. Pregnancy was recorded as premarital if the woman was unmarried or cohabitating at survey time. If a woman was married, we calculated the interval between the end of each pregnancy and the time of marriage. Pregnancies were regarded as post-marital if the interval was greater than eight months, otherwise premarital [25, 40].

#### Regression analysis

A binomial logistic regression model was used to examine the correlation between induced abortions and various factors, including age, residence, ethnicity, education level, and premarital pregnancy experience.

##### Dependent variable

The dependent variable is binary, indicating whether a woman had experienced induced abortion by the time of the survey.

##### Independent variables

The factors included age, residence (urban or rural), ethnicity (Han or minority group), education level (junior middle school or below, high school, and college or above), and whether a pregnancy was premarital. Due to the limited space in the chart, we used a 10-year interval in the models to measure the birth cohort, producing 15–24, 25–34, 35–44, 45–54, and 55–60 cohorts.

We also controlled for potential confounders, including health status, employment status, and number of siblings. These confounders were measured with specificity to the time of the survey rather than the time of the induced abortion.

## Results

### General induced abortion trends

**Fig. 1:**
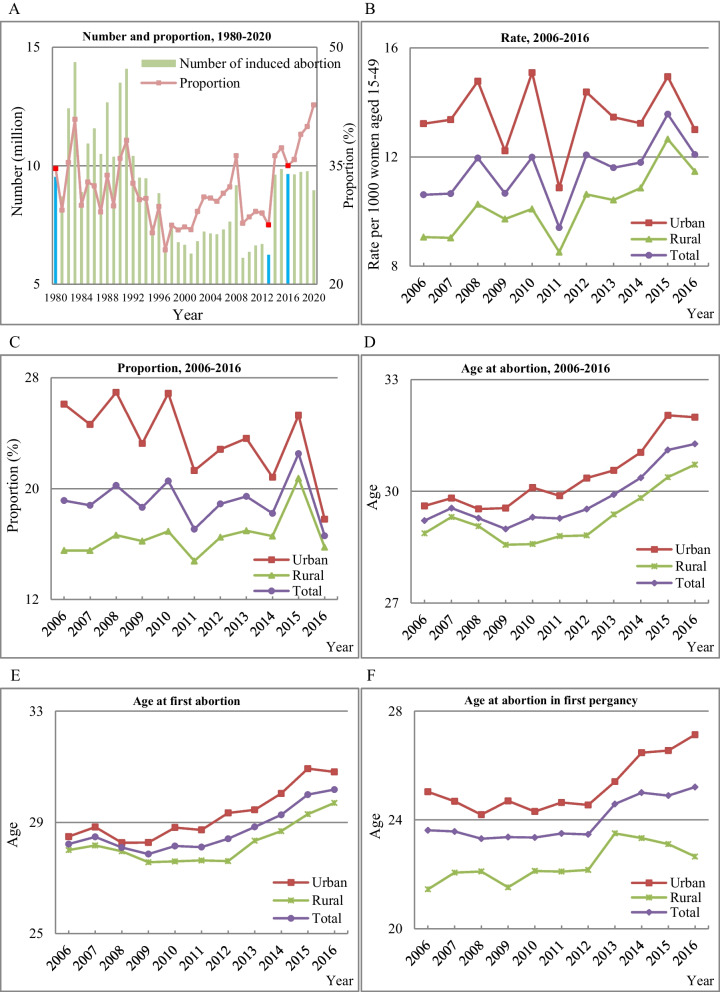
Trend in induced abortion. **A** Number and Proportion, 1980–2020. **B** Rate, 2006–2016. **C** Proportion, 2006–2016. **D** Age at abortion, 2006–2016. **E** Age at first abortion. **F** Age at first pregnancy which ended in abortion. Note: The proportion in Panel 1A is the number of induced abortions/(number of induced abortions + number of births); the research object of Panel 1B-1 F is women of 15–49 years old in the 2017 China Fertility Survey. The years in which the one-child policy (1980), the selective two-child policy (2013) and the universal two-child policy (2016) were introduced are specially marked in Panel 1A

Figure 1 shows the number and proportion of induced abortions, rate of induced abortions, proportion of induced abortions by residence, average age of women undergoing induced abortions, average age when women experience their first induced abortions, and average age of women who had induced abortions during their first pregnancies.

Panel 1A shows that the number and proportion of induced abortions in China fluctuated greatly between 1980 and 2020, and can be divided into three stages. During the first stage (1980–1992), the annual number and proportion of induced abortions remained high. During the second stage (1993–2013), the annual number and proportion of induced abortions declined and remained at a low level. The third stage (2014–2020) was characterized by a return to a higher proportion and number.

Panels 1B and 1C show the rate and proportion of induced abortions, respectively, among women aged 15–49 between 2006 and 2016 in China. The rate fluctuated upward and was higher for urban women than for rural women. The proportion of induced abortions showed a downward trend, reflecting a reduction in unintended pregnancies, especially among urban women.

Panels 1D, 1E, and 1F show the average age of women undergoing induced abortions between 2006 and 2016. Panel 1D indicates an upward trend in age at the time of induced abortion, with urban women tending to be older than rural women. Panel 1E shows that the average age at the time of the first abortion increased from 28.49 to 2006 to 30.81 in 2016 for urban women, and from 28.01 to 29.70 for rural women. Panel 1F presents the average age of the women undergoing first-pregnancy induced abortions. For urban women, this age has increased over the decades, whereas for rural women, it has declined since 2013.

### Age-specific cumulative proportion of induced abortion for a cohort

**Fig. 2:**
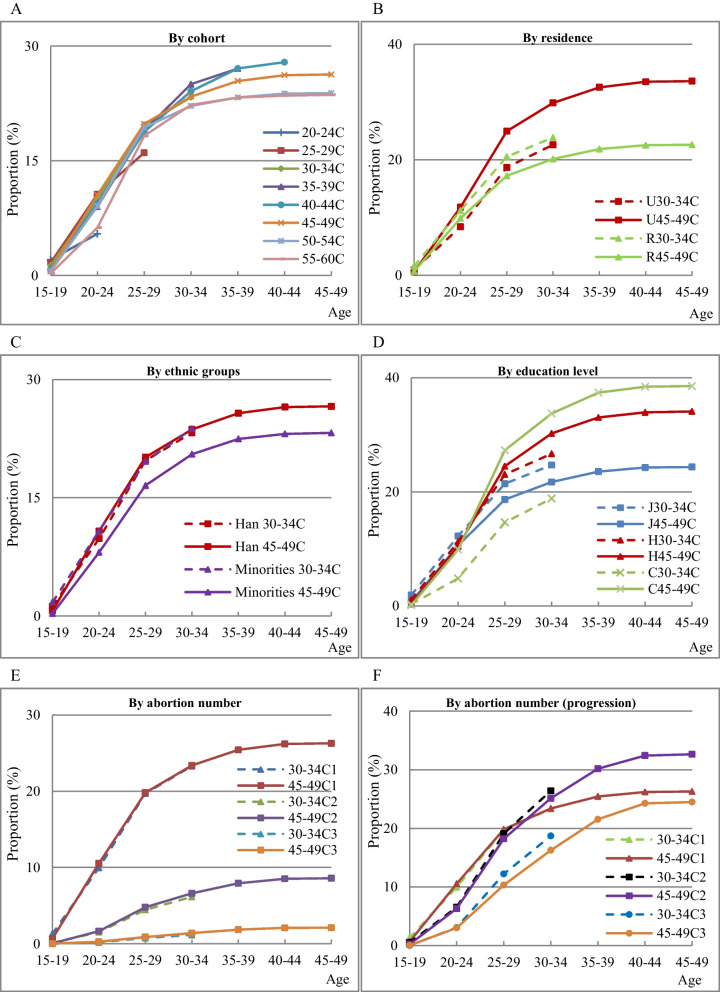
Age-specific cumulative proportion of induced abortion in cohort. **A** By cohort. **B** By residence. **C** By ethnic groups. **D** By education level. **E** By abortion number. **F** By abortion number (progression). Note: C represents cohort. 45-49 C represents the cohort of women aged 45–49 at survey time. U, R for residence, J, H, C for education level. Han and Minorites mean Han women and ethnic minorities women. For example, H30–34C means the cohort of women with a high-school education aged 30–34 at survey time. In Panel 2E, 30–34C2 means women in the 30–34 cohort who have undertaken at least two induced abortion

Figure 2 shows the age-specific cumulative proportion and progression ratio of induced abortions among women who experienced induced abortions by the corresponding age. Panel 2A indicates that less than 30% of the women in different cohorts ever experienced induced abortions.

Panel 2B shows that in the early birth cohort (45–49 years old at survey time), a higher proportion of urban women had experienced an induced abortion by a specific age than rural women. By contrast, in the late birth cohort (30–34 years old at survey time), more rural than urban women had experienced an induced abortion. Among urban women, the proportion of women in the late birth cohort was lower than that in the early birth cohort; however, the data showed a reverse pattern for rural women.

Panel 2C shows that in the early birth cohort, the proportion of Han women who had experienced an induced abortion by a specific age was higher than that of minority women, and this pattern was reversed for the late birth cohort. The proportion of Han women in the late birth cohort was roughly the same as that in the early birth cohort, while the proportion of minority women in the late birth cohort was higher by the corresponding age.

Panel 2D shows that in the early birth cohort, the higher the education level, the higher the proportion of women who experienced induced abortions by the corresponding age. In the late birth cohort, the proportion of women with a high-school education or below was higher than that of women with a college education or above by the corresponding age. Compared with the early birth cohort, the proportion of women with a high-school education or above in the late birth cohort was lower among women with corresponding educational levels by the corresponding age. However, a higher proportion of women with a junior middle-school education or below have experienced induced abortion in the late birth cohort than in the early birth cohort, showing an advance in age at induced abortion among women with lower education levels.

Panel 2E shows the cumulative proportion of women in the cohorts who have experienced a minimum of one, two, or three induced abortions by a specific age. The cumulative proportion of women who experienced at least one induced abortion was 26.29% by the age of 49. Over 70% of women had no abortion experience. Overall, 8.58% of the women in the cohort experienced at least two induced abortions, whereas 2.10% had experienced at least three induced abortions. Panel 2F shows the cumulative proportion of women in the cohort who had experienced one abortion, the progression ratio for women who experienced a second abortion (among women who had experienced a first abortion), and the progression ratio for women who had experienced three abortions (among those who had already had two abortions). Of the women who had experienced one abortion, more than 30% had experienced two or more abortions. Of the women who had experienced two abortions, approximately 25% experienced three or more abortions.

### Age-specific cumulative number of induced abortion

**Fig. 3:**
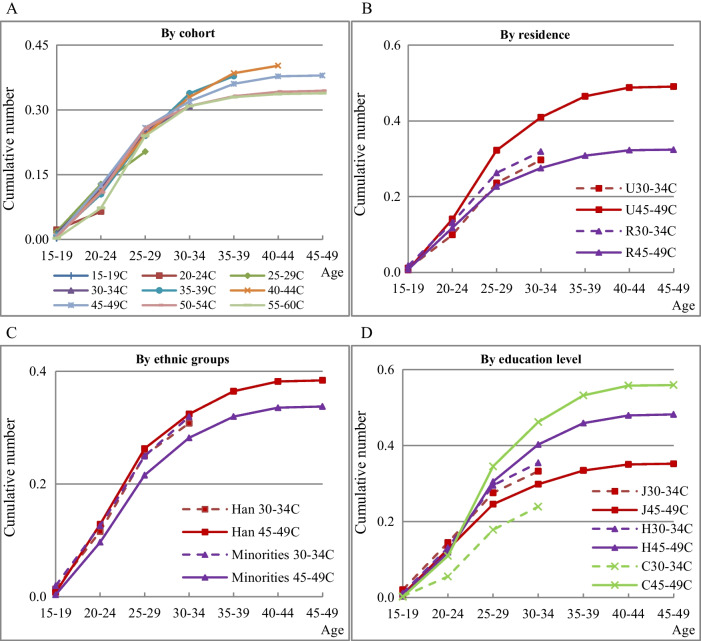
Age-specific cumulative number of induced abortion per woman in cohort. **A** By cohort. **B** By residence. **C** By ethnic groups. **D** By education level Note: C represents cohort. 45–49C represents the cohort of women aged 45–49 at survey time. U, R for residence, J, H, C for education level, as defined in Fig. 2. Han and Minorites mean Han women and ethnic minorities women. For example, H30–34C means the cohort of women with a high-school education aged 30–34 at survey time

Figure 3 shows the age-specific cumulative number of induced abortions per woman in the different cohorts.

Panel 3A shows that the cumulative number of induced abortions per woman is less than 0.45 by the age of 49 in both cohorts. Before the age of 30–34, there was little difference in cumulative numbers. After the age of 30–34, the later the birth cohort, the higher the cumulative number of induced abortions by a specific age.

Panel 3B shows that in the early birth cohorts, the cumulative number of abortions is higher among urban women than among rural women of the same age. However, the opposite is true for late birth cohorts. For urban women, the number for the late birth cohort was lower than that for the early birth cohort by the corresponding age. By contrast, the number for the late birth cohort in rural areas was higher than that for the early birth cohort.

Panel 3C shows that Han women in the early birth cohort had a higher cumulative number of induced abortions than ethnic-minority women, although there was little difference between them in the late birth cohort. Among Han women, the number of induced abortions in the late birth cohort was lower than that in the early birth cohort, whereas the opposite was true among ethnic-minority women.

Panel 3D shows that women with higher education levels in the early birth cohort experienced more induced abortions by a specific age. In the late birth cohort, women with a high-school education or below had more induced abortions than those with a college education or above. Compared with the early birth cohort, women in the late birth cohort with a high-school education or above had fewer cumulative numbers of induced abortions. By contrast, women in the late birth cohort with a junior middle-school education or below had more cumulative numbers of induced abortions than that in the early birth cohort.

### Induced abortions of premarital and unintended pregnancies

**Fig. 4:**
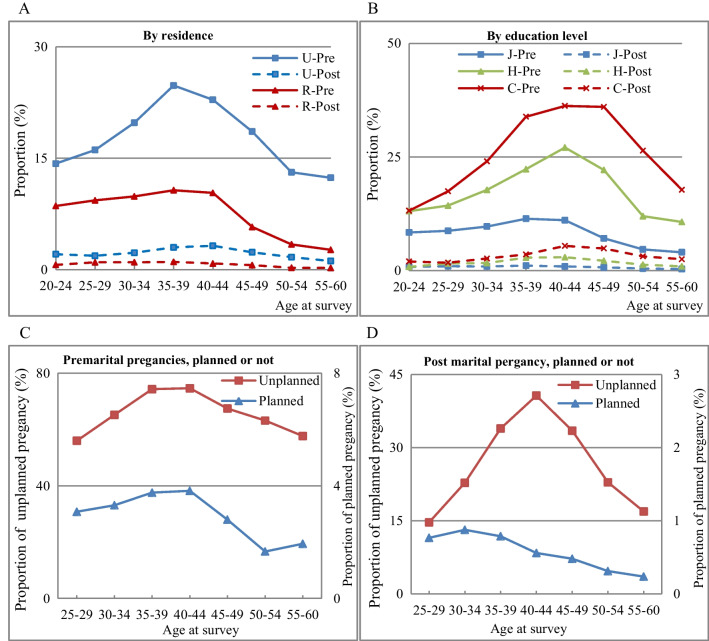
First-pregnancy abortion by birth cohort and marital status. **A** By residence. **B** By education level. **C** Premarital pregnancies, planned or not. **D** Post marital, planned or not. Note: For legends here, U and R represent Urban and Rural; Pre and Post represent premarital or post-marital; J, H, and C represent junior middle school or below, high school, college or above. For example, in Panel 4A, U-Post = Urban women whose first pregnancy is post-marital pregnancy. H-Pre = Women with a high-school education whose first pregnancy is premarital

Figure 4 shows the proportion of first-pregnancy induced abortions by birth cohort and marital status. Regardless of the residence, education level, or whether the pregnancy was planned, more induced abortions occurred before marriage than after.

Panel 4A indicates that regardless of marital status, urban women had a higher proportion of first-pregnancy induced abortions than rural women in both cohorts. Panel 4B shows a positive correlation between education level and first-pregnancy induced abortions. When considering premarital first pregnancies, there was little difference in the proportion of induced abortions among women in both cohorts with a junior middle-school education or below. However, the difference is greater among women with a senior high-school education or above. As Panels 4C and 4D show, the proportion of induced abortions was much higher for unintended pregnancies than for planned pregnancies. The proportion of abortions is much higher for unintended premarital pregnancies than for unintended post-marital pregnancies. The proportion of induced abortions among planned pregnancies was relatively low, both before and after marriage.

### Induced abortions in the next pregnancy by sex of the first child

**Fig. 5:**
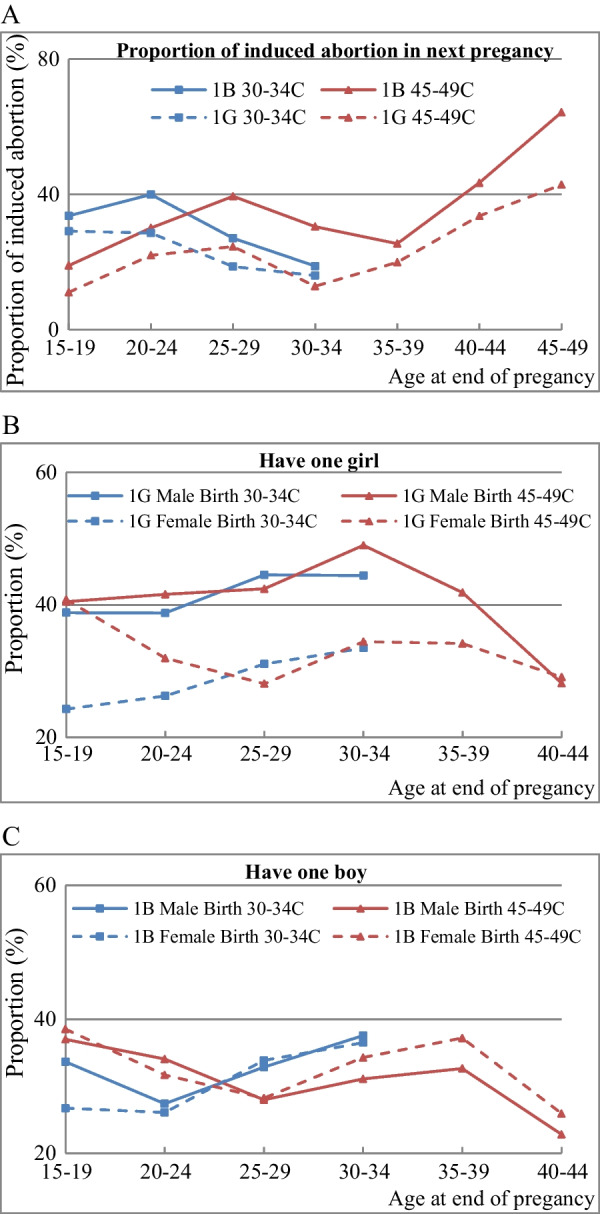
Sex of first child and next pregnancy. **A** Proportion of induced abortion in next pregnancy by sex of the first child. **B** Proportion of male and female births in next pregnancy for a first child of a girl. **C** Proportion of male and female births in next pregnancy for a first child of a boy. Note: 1B 30–34 C represents women in 30–34 cohort whose first child is a boy, 1G 30–34 C represents women in 30–34 cohort whose first child is a girl. 1G Male Birth 30–34 C represents the proportion of male births in the next pregnancy following a first childbirth of a girl in the 30–34 cohort; 1B Male Birth 30–34 C represents the proportion of male births in the next pregnancy following a first childbirth of a boy in the 30–34 cohort

Figure 5 shows the proportion of induced abortions in women’s next pregnancy (following the first childbirth) and the proportions of male and female live births. As Panel 5A shows, more induced abortions occurred following the first birth of a son than following the first birth of a daughter. Panels 5B and 5C show a higher proportion of male births following the first childbirth of a girl, but no significant gender difference following the first childbirth of a boy.

### Logistic regression results

We used a binomial logistic regression model to further examine the correlation between induced abortion experiences and associated factors.

Table 1 shows the percentage distribution of women. Overall, more than 22% of women in this sample had experienced at least one induced abortion at the time of the survey. The lower percentage of women with induced abortion experiences in the 15–24 birth cohort was because of the right censoring. A substantial number of induced abortions did not occur among women in this birth cohort.

**Table 1:** Percentage distribution of women who experienced induced abortions or not

Variables	Not experienced induced abortion		Experienced induced abortion		Total	
	N	%	N	%	N	%
Total	187,721	77.91	53,236	22.09	240,957	100
Cohort						
15–24	25,006	96.94	790	3.06	25,796	100
25–34	39,664	80.20	9795	19.80	49,459	100
35–44	42,372	72.47	16,097	27.53	58,469	100
45–54	62,885	74.92	21,046	25.08	83,931	100
55–60	17,794	76.36	5508	23.64	23,302	100
Residence						
Urban	68,495	74.48	23,469	25.52	91,964	100
Rural	119,226	80.02	29,767	19.98	148,993	100
Ethnic groups						
Han	168,040	77.69	48,246	22.31	216,286	100
Minorities	19,681	79.77	4990	20.23	24,671	100
Education level						
Junior middle school or below	126,641	77.63	36,488	22.37	163,129	100
High school	31,370	76.69	9533	23.31	40,903	100
College or above	29,710	80.46	7215	19.54	36,925	100
Premarital pregnancy or not						
No premarital pregnancy	141,299	81.87	31,285	18.13	172,584	100
Premarital pregnancy	46,422	67.90	21,951	32.10	68,373	100

Table 2 presents the results of the binomial logistic regressions. Model 1 included women’s birth cohort, residence, ethnicity, education level, premarital pregnancy, and other confounders. The results showed that birth cohort, residence, education level, and premarital pregnancy were significantly associated with the experience of induced abortion.

**Table 2:** Results of the binomial regression model for associated factors of induced abortion

	M1		M2	
	AOR	SE	AOR	SE
Cohort (ref. = 15–24)				
25–34	6.813***	0.260	28.805***	3.619
35–44	11.729***	0.443	52.510***	6.516
45–54	10.899***	0.416	60.698***	7.504
55–60	9.924***	0.404	59.362***	7.539
Residence (ref. = Urban)				
Rural	0.687***	0.008	0.866	0.077
Cohort* Residence				
25–34* Rural			0.975	0.091
35–44* Rural			0.874	0.081
45–54* Rural			0.707***	0.065
55–60* Rural			0.619***	0.060
Ethnic group (ref.=Han)				
Minority	0.993	0.017	1.209	0.125
Cohort* Ethnic group				
25–34* Minority			0.903	0.099
35–44* Minority			0.771*	0.083
45–54* Minority			0.758*	0.082
55–60* Minority			0.826	0.099
Education (ref. = Junior middle school or below)				
High school	1.217***	0.018	0.927	0.087
College or above	0.972	0.017	0.616***	0.086
Cohort*Education				
25–34* High school			1.091	0.107
35–44* High school			1.319**	0.129
45–54* High school			1.393**	0.135
55–60* High school			1.502***	0.152
25–34* College or above			1.062	0.151
35–44* College or above			1.993***	0.282
45–54* College or above			2.586***	0.369
55–60* College or above			2.378***	0.387
Premarital pregnancy or not (ref.=No premarital pregnancy)				
Premarital pregnancy	2.115***	0.023	45.612***	4.904
Cohort* Premarital pregnancy or not				
25–34* Premarital pregnancy			0.066***	0.007
35–44* Premarital pregnancy			0.047***	0.005
45–54* Premarital pregnancy			0.035***	0.004
55–60* Premarital pregnancy			0.033***	0.004
Constant	0.027***	0.001	0.006***	0.001

*AOR* adjusted odds ratio, *SE* standard error; ***p < .001; **p < .01; *p < .05; Confounders include health status, employment status, and number of siblings for the women are omitted in the table

**Fig. 6:**
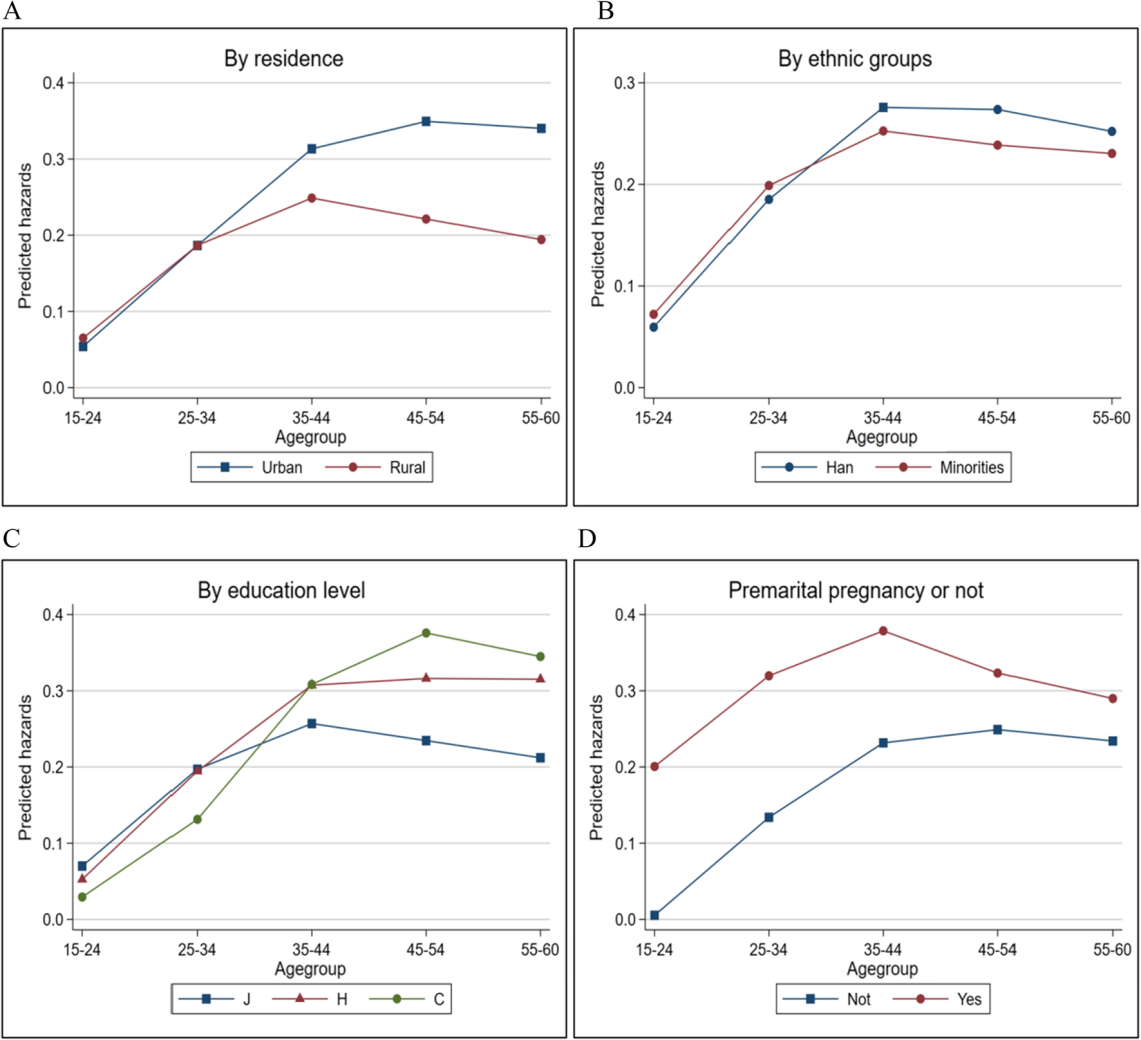
Predicted hazards of experiencing induced abortions. **A** By residence. **B** By ethnic groups. **C** By education level. **D** Premarital pregnancy or not. Note: J, H, and C represent junior middle school or below, high school education, and college or above

Based on Model 1, the interaction terms of birth cohort with residence, ethnicity, education level, and premarital pregnancy were added in Model 2 to examine the differences in the probability of women with different characteristics and across cohorts who experienced induced abortions. Moreover, we calculated the predicted hazards of having an induced abortion by residence, ethnicity, education level, and premarital pregnancy to better interpret the interaction effects in Model 2, as shown in Fig. 6.

Figure 6 presents the average marginal effects of each interaction term based on Model 2 in Table 2. Panel 6A shows that for the earlier birth cohorts (35–44, 45–54, and 55–60), the predicted hazards of induced abortion are higher for women in urban areas than for those in rural areas. However, in later birth cohorts (15–24, 25–34), rural women are more likely to undergo induced abortions, which is consistent with the above results. Panel 6B indicates that in earlier birth cohorts (35–44, 45–54, 55–60), Han women are more likely to experience induced abortions, while in later birth cohorts (15–24, 25–34), ethnic-minority women are more likely to experience induced abortions. Panel 6C shows that in earlier birth cohorts (35–44, 45–54, 55–60), well-educated women are more likely to experience induced abortions, while in later birth cohorts, women with lower education levels are more likely to experience more induced abortions, consistent with the results above. Panel 6D shows that women with premarital pregnancy experience are more likely to undergo induced abortions. Compared with early birth cohorts, women in late birth cohorts with premarital pregnancy experience and those without had a greater difference in the probability of having an induced abortion.

## Conclusion and discussion

This study analyzed the number, rate, and proportion of induced abortions by women’s characteristics and birth cohorts, as well as various correlates and draws the following conclusions:

The number and proportion of induced abortions in China have significantly fluctuated since 1980, and the average age of women undergoing induced abortions increased between 2006 and 2016. From 2006 to 2016, the abortion rate for women aged 15–49 showed an upward trend, while the proportion of induced abortions showed a downward trend. The average age of women undergoing induced abortions showed an upward trend. Rural women were younger than urban women at the time of their first induced abortions. After the Chinese government began to strictly implement its family planning policy in the 1980s, induced abortion became prevalent [41]. Women turned to induced abortion when a pregnancy fell outside the limits established by the government’s birth control policy [22, 36]. Since the mid-1990s, with the introduction of reproductive health and reproductive rights put forward by the Cairo Conference on Human Development in 1994, the Chinese government has promoted informed choice of contraception and birth control, partly reducing the number of unintended pregnancies, and thus the number of induced abortions [42, 43]. Since 2014, the number of induced abortions has rebounded and remained stable. In 2016, when the universal two-child policy was implemented, the number of births increased and the proportion of induced abortions decreased. However, the policy effect disappeared in 2018. The number of births decreased and the proportion of induced abortions rebounded. Regarding age at induced abortion, the number of induced abortions experienced by each woman increases with age; repeat induced abortions occur mainly among women in the older group [12, 17]. Although the minimum age at the time of induced abortion is declining [32, 44], adolescents’ induced abortions still account for a relatively small proportion, contributing to the upward trend in the average age of women having induced abortions.

The proportion of women with induced abortion experiences was less than 30% in each cohort, although this figure changed for women with different characteristics, and the proportion of repeat induced abortions was relatively low. In the early birth cohort, the proportion of women who had experienced induced abortions was higher among urban women than rural women, higher among Han women than ethnic-minority women, and higher among well-educated than less-educated women. This trend is partly due to the fact that women in urban areas and of Han nationality are more tightly constrained by the one-child policy. In the late birth cohort, however, the proportion of women with induced abortion experiences was higher among rural than urban women, and higher among ethnic-minority women than Han women. With the relaxation of the stringent birth control policy, the influences of the one-child policy on induced abortions have been weakened for women in later birth cohorts. And women in urban areas and of Han nationality have greater access to sexual and reproductive health knowledge compared with women in rural areas and of ethnic minority groups, making women in urban areas less likely to undergo induced abortions. In terms of repeat induced abortion, 30.83% of the women in this study had experienced repeat abortions, a lower percentage than that reported in previous research. Many studies have argued that the repeat induced abortion incidence rate is very high in China [18, 19, 35]. A study involving 79,954 women who underwent induced abortion operations in 297 hospitals in 30 provinces in China showed that 65.2% of all induced abortions were repeat induced abortions [19]. Another meta-analysis found that 43.1% of all induced abortions investigated were repeat induced abortions [18]. This may reflect the fact that many studies have been based on small-scale survey data or data collected in hospitals, rather than estimates of nationally representative data.

The cumulative number of induced abortions per woman in each cohort was less than 0.45. In the early birth cohort, the number of abortions was higher for urban than for rural women, for Han than for ethnic-minority women, and for well-educated than for less-educated women. However, in the late birth cohort, these relationships were reversed. Few studies have investigated the number of abortions per woman. One synthetic indicator of the total abortion rate, used to measure the number of abortions a woman would experience if she were to go through her reproductive years experiencing the prevailing age-specific abortion rates, shows a downward trend [5, 9], decreasing from 0.68 to 1993 to 0.37 in 2000 [6].

More induced abortions occur in premarital pregnancies than in post-marital pregnancies. During their first pregnancies, well-educated urban women are more likely to use induced abortion to terminate premarital or unintended pregnancies. In China, the phenomenon of premarital childbearing is not widely accepted, causing most premarital pregnancies to end in induced abortion [25, 45]. One large-scale survey covering 295 hospitals in 30 provinces showed that 31% of more than 70,000 women who underwent induced abortions were unmarried [46]. Women usually choose to give birth when they first become pregnant after marriage; for this reason, the proportion of first-pregnancy induced abortions after marriage is low. The proportion of first-pregnancy induced abortions is higher among urban than among rural women; the higher the level of education, the higher the proportion of induced abortions, and the proportion of induced abortions is much higher in unintended pregnancies than in planned pregnancies.

The sex-selective abortion of female fetuses still exists. When the first child is a girl, the second child is far more likely to be a boy than a girl, indicating that some female fetuses have been aborted. Since the 1980s, with the strict birth control policy and the prevalence of gender-identification technology, the sex-selective abortion of female fetuses has emerged [47]. Sex-selective abortion is one dominant contributor to the higher sex ratio at birth [38, 48, 49]. In the context of a strong preference for sons amid birth-number constraints, the sex ratio at birth for first births was generally normal, but increased steeply for second births, indicating a severe abortion of female fetuses for second and higher birth orders [16]. The stringency of the birth control policy has influenced the extent of sex-selective abortions, as manifested in the provincial sex ratio at birth [50]. With the spontaneous decline in fertility, couples with son preference would selectively abort female fetuses for first births [51], which is confirmed not only in the 2010 census data, when a stringent birth control policy existed, but also by the sex ratio at birth of 113.17 for first births in the 2020 census data, following the introduction of the universal two-child policy in 2016. By contrast, the sex ratio at birth for second births dropped from 130.29 in the 2010 census to 106.78 in the 2020 census as the birth control policy was relaxed.

The finding from the regression analysis showing that age, residence, ethnicity, and education level are significantly associated with women’s induced abortion experiences confirms the findings of previous studies [9, 12, 14, 52, 53], and is consistent with our period and cohort results. The interaction terms in the regression results show that the profile of women who resort to abortion has shifted from well-educated urban women toward rural, less-educated women, contradicting Zheng et al. [9], who found a shift among women resorting to abortion from less-educated rural women to well-educated urban women. They calculated the total abortion rate (the total number of abortions that a woman would experience if she were to go through her reproductive years experiencing prevailing age-specific age abortion rates) based on data from four surveys in 1988, 1997, 2001, and 2006, which are earlier than data we used in this study. In addition, the group more likely to undergo induced abortions has shifted from Han to minority women, a result seldom mentioned in previous studies [9, 12, 14, 17]. Premarital pregnancies are more likely to end in abortion, which is consistent with previous studies [20, 25].

In summary, the above results reveal several problems requiring policy attention. The first is the increasing number of premarital pregnancies among young women, most of which end in abortion. The second is repeat induced abortion, which highlights the need to reduce the incidence of unintended pregnancies [19, 35]. The third is the existence of sex-selective abortion of female fetuses in China, although the Chinese government has made efforts to combat sex identification and sex-selective abortion for non-medical reasons. Optimistically, the decline in the recent sex ratio at birth indicates a waning preference for sons. To prevent and reduce unwanted pregnancies and abortions, the government should implement more effective measures to promote sexual and reproductive health education among young people, provide higher-quality contraceptive and post-abortion care services, and crack down on sex-selective abortions.

This study has some limitations. First, the pregnancy histories used in this study were drawn from retrospective data, which may include underreporting and recall bias, especially among women in the early birth cohort [9, 25]. Despite this, the survey data do reflect the overall levels and changing trends of induced abortion. Second, the survey recorded each woman’s education level at the time of data collection, not at the time of her induced abortion. As most people finish school before marriage and childbearing [25], we assume that the education level is unlikely to change significantly. However, some premarital young women with premarital pregnancies may continue to receive more education. Third, given the lack of data on unsafe abortions, we did not include analysis in this regard. We call on future surveys to consider the prospect of expanding data collection to allow for the analysis of unsafe abortions.

## Data Availability

The datasets generated and analyzed during the current study are not publicly available due to privacy or ethical restrictions but are available from the corresponding author on reasonable request.

## References

[1] Sedgh G, Henshaw S, Singh S, Ahman E, Shah IH (2007). Induced abortion: estimated rates and trends worldwide. Lancet.

[2] Bearak J, Popinchalk A, Ganatra B (2020). Unintended pregnancy and abortion by income, region, and the legal status of abortion: estimates from a comprehensive model for 1990–2019. Lancet Glob Health.

[3] Sedgh G, Bearak J, Singh S (2016). Abortion incidence between 1990 and 2014: global, regional, and subregional levels and trends. Lancet.

[4] National Health Commission (2020). China health statistics yearbook.

[5] Wu S, Qiu H (2010). Induced abortion in China: problems and interventions. Acta Acad Med Sin.

[6] UNFPA. SEEING THE UNSEEN: The case for action in the neglected crisis of unintended pregnancy. 2022. https://www.unfpa.org/sites/default/files/pub-pdf/EN_SWP22%20report_0.pdf. Accessed 15 June 2022.

[7] Fang J, Tang S, Tan X, Tolhurst R (2020). Achieving SDG related sexual and reproductive health targets in China: What are appropriate indicators and how we interpret them?. Reprod Health.

[8] Tan X, Fang J, Xiao C, Liao A, Gong X (2019). The situation and countermeasure of induced abortion and contraceptive of China in the context of sustainable development goals of UN. Chin J Fam Plan.

[9] Zheng X, Pang L, Tellier S (2013). The changing patterns of abortion among married women in China, 1984–2005. Eur J Obstet Gyn R B.

[10] Wang C (2014). Induced abortion patterns and determinants among married women in China: 1979 to 2010. Reprod Health Matter.

[11] Wang C (2017). The impact of the state’s abortion policy on induced abortion among married women in China: a mixed methods study. Chin Sociol Rev.

[12] Tang L, Wu S, Liu D, Temmerman M, Zhang W (2021). Repeat induced abortion among Chinese women seeking abortion: two cross sectional studies. Int J Env Res Pub He.

[13] Stone N, Ingham R (2011). Who presents more than once? Repeat abortion among women in Britain. J Fam Plan Reprod H.

[14] Zhang B, Nian Y, Palmer M (2018). An ecological perspective on risk factors for repeat induced abortion in China. Sex Reprod Healthc.

[15] Gu B, Wang F, Guo Z (2007). China’s local and national fertility policies at the end of the twentieth century. Popul Dev Rev.

[16] Jiang Q, Zhang C (2021). Recent sex ratio at birth in China. BMJ Glob Health.

[17] Wei Z, Yu D, Liu H (2020). Trends and characteristics of induced abortion among married women of reproductive ages in China: a study based on 1997–2017 China fertility surveys. Popul Res.

[18] Liu J, Duan Z, Zhang H (2021). Prevalence and risk factors for repeat induced abortion among chinese women: a systematic review and meta-analysis. Eur J Contracep Repr.

[19] Luo H, Wu S, Wang K (2021). Repeat induced abortion in 30 chinese provinces: a cross-sectional study. Int J Gynecol Obstet.

[20] He H, Blum RW (2013). Prevalence of unintended pregnancy and its associated factors among sexually active never-married youth in Shanghai. J Paediatr Child H.

[21] Guo C, Pang L, Wen X, Zheng X (2019). Risky sexual behaviors and repeat induced abortion among unmarried young women in China: results from a large, nationwide, population-based sample. J Womens Health.

[22] Basten S, Jiang Q (2014). China’s family planning policies: recent reforms and prospects. Stud Family Plann.

[23] Zeng Y, Hesketh T (2016). The effects of China’s universal two-child policy. Lancet.

[24] Jing W, Liu J, Ma Q, Zhang S, Li Y, Liu M (2022). Fertility intentions to have a second or third child under China’s three-child policy: a national cross-sectional study. Hum Reprod.

[25] Qian Y, Jin Y (2020). Premarital pregnancy in China: cohort trends and educational gradients. Stud Family Plann.

[26] Olukoya AA, Kaya A, Ferguson BJ, Abouzahr C (2001). Unsafe abortion in adolescents. Int J Gynaecol Obstet.

[27] Wu S, Tian L, Xu F (2011). Induced abortion and relevant factors among women seeking abortion in Nanjing, China. Gynecol Obstet Inves.

[28] Huang D (2016). Unwanted pregnancy among unmarried youth in mainland China: a literature review. Contemp Youth Res.

[29] Chan KW, Zhang L (1999). The “hukou” system and rural-urban migration in China: processes and changes. China Quart.

[30] Wu X, Treiman DJ (2004). The household registration system and social stratification in China: 1955–1996. Demography.

[31] OECD. SF2.4: share of births outside of marriage. 2018. https://www.oecd.org/els/family/SF_2_4_Share_births_outside_marriage.pdf. Accessed 20 May 2022.

[32] Yu J, Xie Y (2021). Recent trends in the chinese family: national estimates from 1990 to 2010. Demogr Res.

[33] Zhou Y (2018). The dual demands: gender equity and fertility intentions after the one-child policy. J Contemp China.

[34] Lou C, Zuo X, Yin X (2016). Psychological status, barriers and help-seeking behaviors during decision-making for induced abortion among unmarried pregnant women in three cities of China. Chin J Publ Heal.

[35] Liu J, Wu S, Xu J, Temmerman M, Zhang W (2017). Repeat abortion in chinese adolescents: a cross-sectional study in 30 provinces. Lancet.

[36] Qiao J, Wang Y, Li X (2021). A lancet commission on 70 years of women’s reproductive, maternal, newborn, child, and adolescent health in China. Lancet.

[37] Chu J (2001). Prenatal sex determination and sex-selective abortion in rural central China. Popul Dev Rev.

[38] Fan S, Xiao C, Zhang Y, Li Y, Wang X, Wang L (2020). How does the two-child policy affect the sex ratio at birth in China? A cross-sectional study. BMC Public Health.

[39] Zhuang Y, Yang S, Qi J, Li B, Wang Z (2018). 2017 China Fertility Survey: practices and reflections. Popul Res.

[40] Raymo JM, Iwasawa M (2008). Bridal pregnancy and spouse pairing patterns in Japan. J Marriage Fam.

[41] White T (2006). China’s longest campaign-birth planning in the people’s Republic, 1949–2005.

[42] Liu H, Wei Z (2006). The relationship between the informed choice of contraceptive methods and induced abortion. Collect Women Stud.

[43] China Family Planning Association, China Population and Development Research Center (2020). Report on reproductive health in China.

[44] Liu J, Wu S, Xu J (2019). Is repeat abortion a public health problem among Chinese adolescents? A cross-sectional survey in 30 provinces. Int J Env Res Public Health.

[45] Luo D, Yan X, Xu R (2020). Chinese trends in adolescent marriage and fertility between 1990 and 2015: a systematic synthesis of national and subnational population data. Lancet Glob Health.

[46] Wu S, Temmerman M, Wang K, Wang S, Li J, Zhang W (2015). Induced abortion in 30 chinese provinces in 2013: a cross-sectional survey. Lancet.

[47] Zeng Y, Tu P, Gu B, Xu Y, Li B, Li Y (1993). Causes and implications of the recent increase in the reported sex ratio at birth in China. Popul Dev Rev.

[48] Hesketh T, Lu L, Xing ZW (2005). The effect of China’s one-child family policy after 25 years. New Engl J Med.

[49] Jha P, Kesler MA, Kumar R (2011). Trends in selective abortions of girls in India: analysis of nationally representative birth histories from 1990 to 2005 and census data from 1991 to 2011. Lancet.

[50] Zeng Y (2007). Options for fertility policy transition in China. Popul Dev Rev.

[51] Jiang Q, Li Y, Sánchez-Barricarte JJ (2016). Fertility intention, son preference, and second childbirth: survey findings from Shaanxi province of China. Soc Indic Res.

[52] Jones R, Jerman J, Ingerick M (2018). Which abortion patients have had a prior abortion? Findings from the 2014 U.S. abortion patient survey. J Womens Health.

[53] Lindberg L, Kost K, Maddow-Zimet I, Desai S, Zolna M (2020). Abortion reporting in the United States: an assessment of three national fertility surveys. Demography.

